# Supplementary material for: Splice-Junction-Based Mapping of Alternative Isoforms in the Human Proteome
Source: Cell Rep. Author manuscript; Available in PMC 2020 Jan 15. (PMC6961840; doi:10.1016/j.celrep.2019.11.026)
Supplement: 2 [file NIHMS1546469-supplement-2.zip › DF1/PXD010154/Colon/Colon_16_LARP4B_DGQFVSTSNVAEPQTQR.pdf]

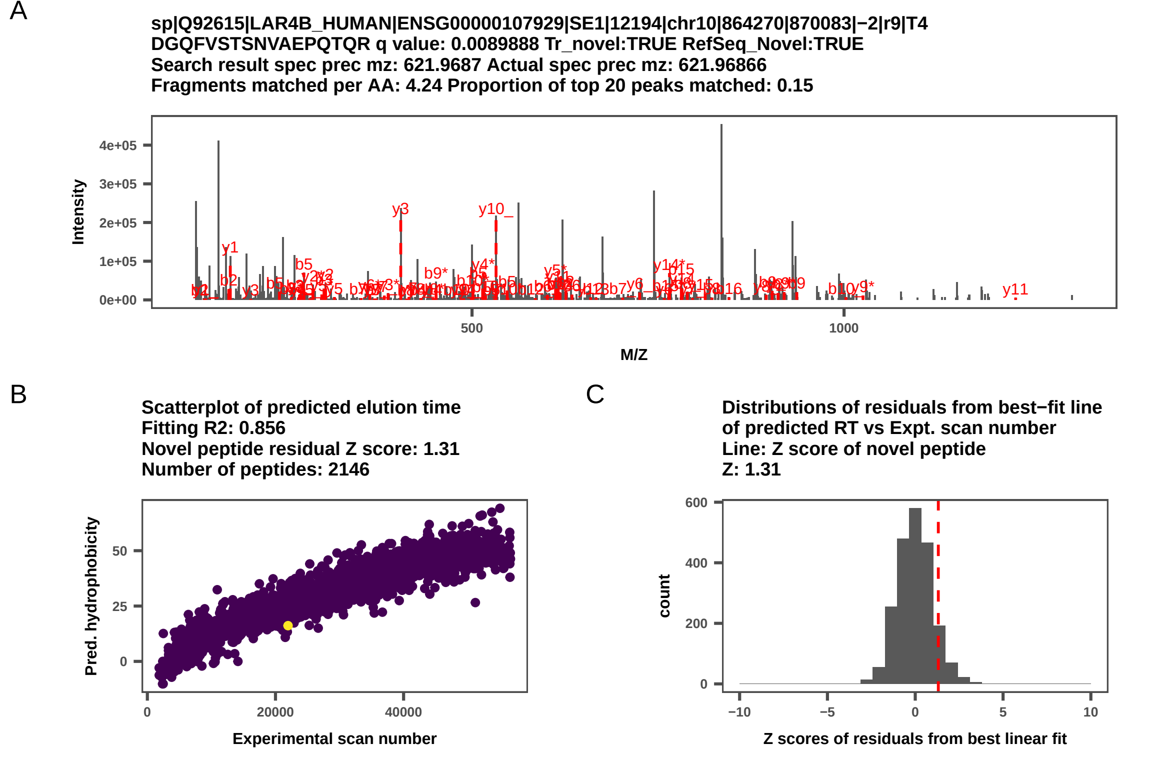

A
sp|Q92615|LAR4B_HUMAN|ENSG00000107929|SE1|12194|chr10|864270|870083|−2|r9|T4
DGQFVSTSNVAEPQTQR q value: 0.0089888 Tr_novel:TRUE RefSeq_Novel:TRUE
Search result spec prec mz: 621.9687 Actual spec prec mz: 621.96866
Fragments matched per AA: 4.24 Proportion of top 20 peaks matched: 0.15
Intensity
4e+05
3e+05
2e+05
1e+05
0e+00
y3
y10_
y1
b2
b5
b9*
y4*
y14*
b15
y11
500
1000
M/Z
B
Scatterplot of predicted elution time
Fitting R2: 0.856
Novel peptide residual Z score: 1.31
Number of peptides: 2146
Pred. hydrophobicity
50
25
0
20000
40000
Experimental scan number
C
Distributions of residuals from best−fit line
of predicted RT vs Expt. scan number
Line: Z score of novel peptide
Z: 1.31
count
600
400
200
0
−10
−5
0
5
10
Z scores of residuals from best linear fit
